# Prosthesis

**Published:** 1898-06

**Authors:** Wm. Ernest Walker


ARTICLE III.

### PROSTHESIS.

WM. ERNEST WALKER, D. D. S.

Proceed as follows: Having secured an accurate plaster-cast, coat it with rubber cement (amber cement furnished by the Chase Combination Dental Plate Company is excellent for this purpose), Press on to the cast a sheet of vulcanizable rubber, to which it will adhere because of the rubber cement. When pressing the rubber on to the cast, if there are any decided depressions on the alveolar ridge, they may be filled with small pieces of rubber before the large piece is put over all, which will give a uniform surface. This is now vulcanized, but only about one-half or two-thirds the usual time. This gives a vulcanit base-plate which will fit more accurately than it would if made by any other method, as the case escapes the usual screw pressure which obliterates the finer features of the cast, and which occasionally results in fracture of the cast, as shown by the sharp thread-like ridge which is often found crossing the vulcanized plates.

This is now used as a base-plate in obtaining the occlusion, or "bite," when it is filled with plaster and placed on the articulator. To this vulcanit base-plate the teeth are now waxed, and the plate is found to be much more steady and more satisfactory while trying in than if only a wax plate had been used. Another advantage in this method is that the ruge are preserved; and if it is found that the plate does not fit, we can take another impression, and thus save the time occasionally lost in setting up the teeth when the impression is faulty. The gums may now be carved up and plate finished in the usual way; or at this stage the following method may be adopted: Having waxed the teeth on the vulcanit base-plate and tried them in, remove any wax from between the teeth, which can be done with chloroform. Now, instead of carving up the gums, lay a strip of moldin on the gum at the necks of the teeth; punch holes in a semi-circular piece of very thin rubber-dam, and pass the teeth through, stretching the rubber over the moldin, which, by a little manipultation through the rubber, will form a very natural gum. When and where necessary the dam can be turned back and moldin added, and again pressed to shape under the rubber-dam. After flasking, the dam is pulled out and the moldin brought with it, leaving the plaster clear.

Another great time-saving method, which can be used in some cases, is as follows: Having removed the bite-wax from the vulcanit base-plate, clear the surface of the latter with chloroform, benzine or gasoline. The alveolar ridge portion is next coated with amber cement and covered with a sheet of rubber, not extending it over the plate. The teeth are now warmed and pressed into the rubber; when it will be found that the rubber bulges over their necks, forming a very natural festoon, without any spatulation. Teeth with a slight groove at the neck, such as are made for celluloid work, are best for this method. In trying the piece in the mouth, any desired change in the position of the teeth is made by removing the tooth, warming and resetting. When the teeth are satisfactorily arranged, a piece of modeling composition is used to fill the vault to support the teeth from the lingual side.

When the composition is hard the teeth are removed and a thin sheet of vulcanizable pink rubber is warmed and placed over the labial and buccal surface, extending over the depressions in the red rubber from which the teeth have been removed, and into which the pink rubber sinks and shows nicely where each tooth is to be placed. Each tooth is now warmed and replaced, the impression in the compound serving to guide each tooth into the correct position. The piece is now flasked on the cast and plaster built up so as to support the buccal and labial faces of the teeth.

When the plaster is hard the modeling composition is removed. Small pieces of rubber are now warmed and placed about the pins so as to anchor the teeth, which must first be coated with amber cement.

The Griswold flask is excellent for this work, as it supports the plaster which surrounds the teeth, but an ordinary flask will do. No separating fluid is necessary before filling the upper portion of the flask, as it will not have to be opened till vulcanized. No allowance of time is to be made for the previous vulcanization of the base-plate. When the piece is removed the usual excess is not found, and after a little trimming at edges of plate and overpins, attention can be given to labial surface, which is found to require no scraping, but should be cleaned with pumice carried by bibulous paper moistened with water or alcohol, or, best of all, chloroform, and then with chalk on chamois or flannel; after which the color of the gums is improved by placing in a glass dish of alcohol in the sun, which bleaches it.

Note that the time usually spent in "waxing up" is saved; also the time usually spent in waiting for the plaster to harden that the flask may be opened; likewise that ordinarily consumed in washing out wax, packing and closing flask, as well as that generally spent in scraping and filing, while an even more important consideration being that the hard, vulcanized surface of the rubber is preserved most all over the plate. The ruge are also well represented, as the rubber over that portion is uniform in thickness.—*Plugger Points.*

